# Potential plant leaves as sustainable green coagulant for turbidity removal

**DOI:** 10.1016/j.heliyon.2023.e16278

**Published:** 2023-05-15

**Authors:** Ayat Khalid Salem, Asia Fadhile Almansoory, Israa Abdulwahab Al-Baldawi

**Affiliations:** a Department of Ecology, College of Science, University of Basrah, Basrah, Iraq; b Department of Biochemical Engineering, Al-khwarizmi College of Engineering, University of Baghdad, Baghdad, Iraq

**Keywords:** Local plant, Biomass, Coagulant, Purification, Kaolin

## Abstract

Chemical coagulation–flocculation has been used widely in water and wastewater treatment. In the present study, green coagulant was investigated. The role of Iraqi plants was examined to remove turbidity by using kaolin synthetic water. Thirteen selected plants were prepared as powdered coagulant. The experiment was run based on coagulant mass varied from 0 to 10,000 mg/L for each plant with a rapid mixing speed of 180 rpm for 5 min, slow mixing speed at 50 rpm for 15 min and settling time for 30 min. The seven best green coagulants are *Albizia lebbeck* (L.), *Clerodendrum inerme* (10,000 mg/L), *Azadirachta indica*, *Conocarpus lancifolius*, *Phoenix dactylifera* (5000 mg/L), *Dianthus caryophyllus* (3000 mg/L) and *Nerium oleander* (1000 mg/L) with turbidity removal rates of 39.3%, 51.9%, 67.2%, 75.5%, 51.0%, 52.6% and 57.2%, respectively. The selected seven plants that were used as green coagulants are economically feasible to achieve the highest turbidity and removal of other compounds.

## 1 Introduction

The increase in population should be considered in dealing with the requirements of clean water demands, therefore need to think of ways to water treatment sustainable. Wastewater enters surface water caused by the pollution of rivers and lakes with organic and inorganic compounds, and this water cannot be directly used [1,2]. Consequently, before being used or discharged, the river water and wastewater should be treated to remove pollutants before distribution [3]. Turbidity is usually used for the evaluation of water and wastewater quality [4]. The wastewater from different industrial and agricultural manufacturers contains various pollutants with colloidal and suspended particles, such as organic compounds, heavy metals, dyes and pesticides, which need to be pretreated. One of the physicochemical technologies for wastewater treatment is coagulation/flocculation, which can be used as efficiently for the treatment of wastewater [5,6].

Coagulation–flocculation involves the removal of suspended solid and colloids particles in water and wastewater by destabilization and aggregation the particles into larger aggregates. The settled aggregates can be quickly and simply separated from the water [7]. Green coagulant is an improved coagulation technology for essential treatment processes to remove suspended solids in water treatment plants with safe residual.

The coagulation–flocculation process is an important step in wastewater treatment because of its removal efficiency for suspended solid and colloids particles. Chemically enhanced primary treatment uses metallic coagulants such as alum (AlCl_3_), ferric chloride (FeCl_3_), polyaluminium chloride, ferric chloride and ferrous sulphate for the removal of pollutants traditionally. The use of chemical agent has many disadvantages, such as pH variation, alkalinity addition and higher dosage.

The utilization of chemical coagulants can lead to produce non-biodegradable sludge (Maurya1 and Daverey, 2018). Therefore, many studies have been investigated and concluded to adopt biomass of plants or bacteria as coagulants because it is environmentally friendly and economical [8,9].

Green coagulation–flocculation has been studied, and good results were obtained in the pre-treatment of potable water and wastewater from oily, metal, food, dyeing and municipal industries [[9], [10], [11], [12], [13]]. [14] used *Moringa oleifera* Lam seeds extracted with KCl for the coagulation–flocculation process of textile wastewater and reached a removal rate of 82.2% for the apparent colour. Other researchers have tested the performance of plant extraction methods with salt (NaCl), alkaline (NaOH) and acid (HCl), thus improving the adsorption ability and flocculation advantage of green coagulants and increasing pollutant removal efficiency [15]. [16] extracted *Avicennia marina* plants with hydrochloric acid, sodium hydroxide and sodium chloride and obtained better turbidity removal performance than the native *Avicennia marina* coagulant.

The biocoagulants/bioflocculants extracted from plants leaves, animal and microorganisms have positive charges, whereas the suspended solids or colloids in water and wastewater are almost negatively charged [3]. The compounds of polysaccharides, protein polymers and some functional groups, such as hydroxyl and carboxyl groups structured in biocoagulants/bioflocculants play an important role in natural coagulants [17].

The advantages of the use of green coagulation–flocculation are the recovery and recycling of sludge from primary treatment for their possible use in fertilization for non-toxic wastewater [18]. In coagulation–flocculation, many studies have been conducted in the treatment of municipal, institutional dairy, agriculture, aquaculture and grey wastewater [7,9,[19], [20], [21], [22]].

Table 1 summarises various findings on the use of green coagulants as sustainable coagulants, which are environment-friendly coagulants for water and wastewater treatment [18]. investigated the recovery of microalgae by using green coagulants of *Moringa oleifera* seed (pH 4 and 40 mg/L dose) and *Guazuma ulmifolia* barks (pH 7 and 30 mg/L dose) with recovery efficiencies of 80% and 60%, respectively.

**Table 1:** Research on green coagulation application.

Industry	Source of coagulant	Conditions	Removal efficiency	Reference
Recovery of microalgae	*Moringa oleifera* seed *Guazuma ulmifolia* barks	pH 4, dose 40 mg/L pH 7 dose 30 mg/L	80%, 60%	[18]
Institutional wastewater	Cassava peel starch	pH 6.0, dose 448.58 mg/L	60.19% for turbidity, 57.79% for TSS, and 30.19% for COD	[20]
Dairy wastewater	Okra Passion fruit seeds	pH 9/Dose 2 g/L pH 5/Dose 1.3 g/L	Turbidity: 91.1% COD: 48.3% Turbidity: 91.5% COD: 50.3%	[9]
Dairy industry	*Cicer arietinum* *Moringa oleifera* *Strychnos potatorum*	pH 6.3/Dose 400 mg/L pH 6.2/Dose 400 mg/L pH 6.4/Dose 800 mg/L	Turbidity: 74.23% BOD: 63.2% Turbidity: 65.6% BOD: 70.9% Turbidity: 75.62% BOD: 72.1%	Deepa et al. (2022)
Municipal Wastewater	Banana peel powder, Banana stem juice, Papaya seed powder and neem leaf powder	Dose 0.4 g/L, pH 7-8 Dose. 10 mL/L, pH 7-8 Dose 0.8 g/L, pH 7-8 Dose 1 g/L, pH 7-8	Turbidity, TSS, and COD 59.6%, 45.45, and 58% 18.78, 29.69, and 64% 41.89, 66.67, and 66.67% 43.96, 35.75, and 64%	[19]
Industrial Wastewater	Egg shells	Dose 20 mg/L 150 rpm for 2 min and 30 rpm for 15 min	Turbidity 98.89%, color 98.89%, and TSS 98.52%	[23]
Agro Industrial Wastewater	*Acacia dealbata* leaf	pH 3.0	Turbidity, 84.7 Total suspended solids (TSS) 79.1, and Volatile suspended solids (VSS) 76.6%,	[21]
Drinking Water Quality	Aloe vera	pH 7	Turbidity 87.84%	[24]
Grey wastewater	*Moringa oleifera*	Dose 50 g/L pH 7.2 to 6.8	Turbidity 96.22%	[7]

Green coagulant is an interesting alternative tool in developing countries such as Iraq for small treatment plants, because it is economical, eco-friendly and has low toxicity of residual sludge. In the present work, 13 Iraqi tree leaves were used as a novel and promising green coagulant for the treatment of wastewater after observed with kaolin removal. The selected plants are prevalent in Iraq with four seasons, and their leaves can be harvested throughout the year. These plants can also be found in other countries of similar weather. The coagulant was extracted from local dry green leaves by using water as a safe solution without adding chemical for primary wastewater treatment in industry and agriculture wastewater effluent. Therefore, this study aimed to determine the best leaves among these 13 plants that can act as effective natural green coagulants for turbidity removal. The sustainability aspects of the green coagulants were determined according to the conditions of jar test run.

## 2 Materials and methods

### 2.1 Plant selection and coagulant powder

Thirteen local plants were chosen to determine whether they can be used as green coagulants. Table 2 consists of 13 selected local plants of *Acacia greggii*, *Albizia lebbeck* (L.), *Aloe barbadensis, Azadirachta indica*, *Bougainvillea glabra*, *Clerodendrum inerme*, *Conocarpus lancifolius, Dianthus caryophyllus*, *Eucalyptus camaldulensis*, *Eucalyptus citriodora*, *Moringa oleifera*, *Nerium oleander* and *Phoenix dactylifera*. First, plant leaves were collected and washed with distilled water, and then placed in an oven under 70 °C for 2 days. The dried leaves were ground and sieved to obtain a fine powder with 38 μm, and the sample was kept in a closed container [25] to be used for coagulant water extraction. Water extraction was adopted to prepare a stock solution of 500–10,000 mg/L coagulants by adding 10 g of plant powder to 100 mL of distilled water, and then mixing them for 30 min [20]. The coagulant extraction was filtered through a 0.33 mm ZELPA Belgium paper and kept in a Duran bottle. These steps of coagulant extraction were carried out at the same time.

**Table 2:** Local selected plants for the preparation of coagulants.

Plant scientific name	Plant common name	Full plant Profile	Plant Leaves
*Acacia greggii*	Catclaw acacia		
*Albizia lebbeck* (L.)	Silk trees		
*Aloe barbadensis*	Aloe vera		
*Azadirachta indica*	Neam		
*Bougainvillea glabra*	Bougain villea		
*Clerodendrum inerme*	Blue Jasmine		
*Conocarpus lancifolius*	Buttonwood		
*Dianthus caryophyllus*	Carnation		
*Eucalyptus camaldulensis*	Red river gum		
*Eucalyptus citriodora*	Lemon-scented gum		
*Moringa oleifera*	Moringa		
*Nerium oleander*	Oleander		
*Phoenix dactylifera*	Palm		

### 2.2 Water with kaolin preparation

For test plant as green coagulant, synthetic turbid water was prepared by mixing Kaolin powder with water. A 500 NTU concentration of synthetic turbid water was prepared by mixing kaolin (1.5 g) with distilled water (3 L) in beaker, and the mixture was stirred for 30 min to be homogeneous to simulate an initial turbidity concentration of 500 ± 50 NTU [25,26].

### 2.3 Procedure for testing green coagulant removal efficiency

The test was carried out using jar test with six 1 L beakers (Simax, Czech Republic) with working volume of 500 mL. Water plant extract was prepared by adding different mass dosages of each plant into six beakers with coagulant concentrations of 0 mg/L (control), 500, 1,000, 3,000, 5000 and 10,000 mg/L. High concentrations of natural coagulants are required because of their weak coagulation capability [27]. The test was adopted at a rapid mixing speed of 180 rpm for 5 min and a slow mixing speed of 50 rpm for 15 min by jar test [28]. After the two options of rapid and slow mixed of coagulation-flocculation process, the suspended solid was left to settle for 30 min. Approximately 10 mL of sample from the top of each beaker was collected to read their turbidity by using a turbidity meter (Lovibond, Germany). The turbidity removal efficiency was determined using Equation (1) [4]. Three samples were obtained from each beaker to decrease the error in reading and average value calculated.

(1) $$Turbidity\;removal\;from\;water\;\left(\%\right)=\frac{{Tu}_{i}-{Tu}_{f}}{{Tu}_{i}}\times 100$$

where *Tu*_*i*_ and *Tu*_*f*_ (NTU) are the initial and final turbidity in water, respectively.

### 2.4 Statistical analysis for turbidity removal efficiency

The removal efficiency of turbidity by green coagulant was statistically analyzed using SPSS Software version 21 (IBM, USA) for confidence of 95% at p < 0.05 to represent a significant difference from the results taken from the experiments. One-way ANOVA was used to determine the effect of different green coagulant mass on turbidity removal by post hoc test by using Turkey HSD [29].

## 3 Results and discussion

### 3.1 Response of green coagulants on turbidity removal

**Fig. 1:**
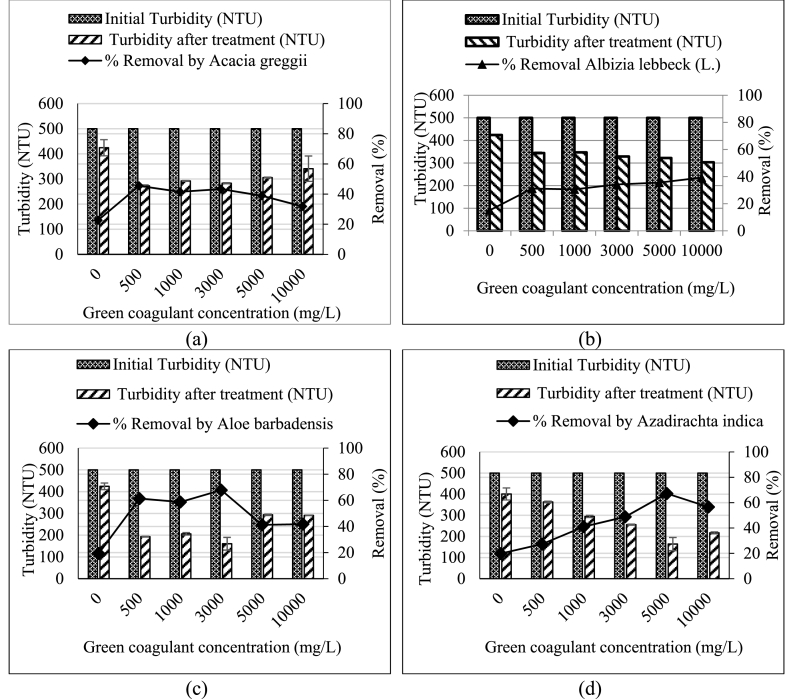
Role of the selected plants for turbidity removal with different coagulant dosages compared with the 500 NTU turbidity without green coagulant as control (0 g/L): (a) *Acacia greggii*, (b) *Albizia lebbeck* (L.) (c) *Aloe barbadensis* (L.), and (d) *Azadirachta indica*.

**Fig. 2:**
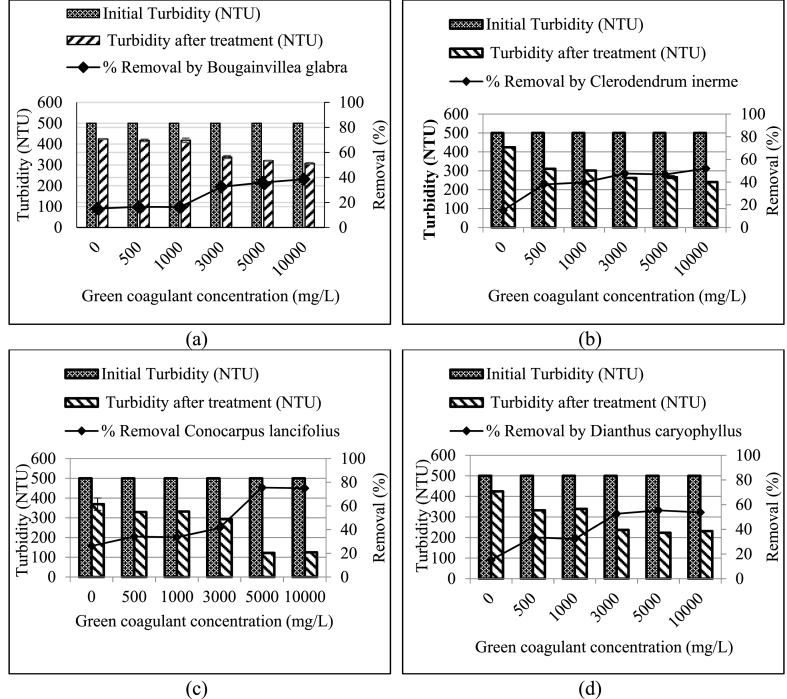
Role of selected plants for turbidity removal with different coagulant dosages compared with the 500 NTU turbidity without green coagulant as control (0 g/L): (a) *Bougainvillea glabra*, (b) *Clerodendrum inerme*, (c) *Conocarpus lancifolius* and (d) *Dianthus caryophyllus*.

**Fig. 3:**
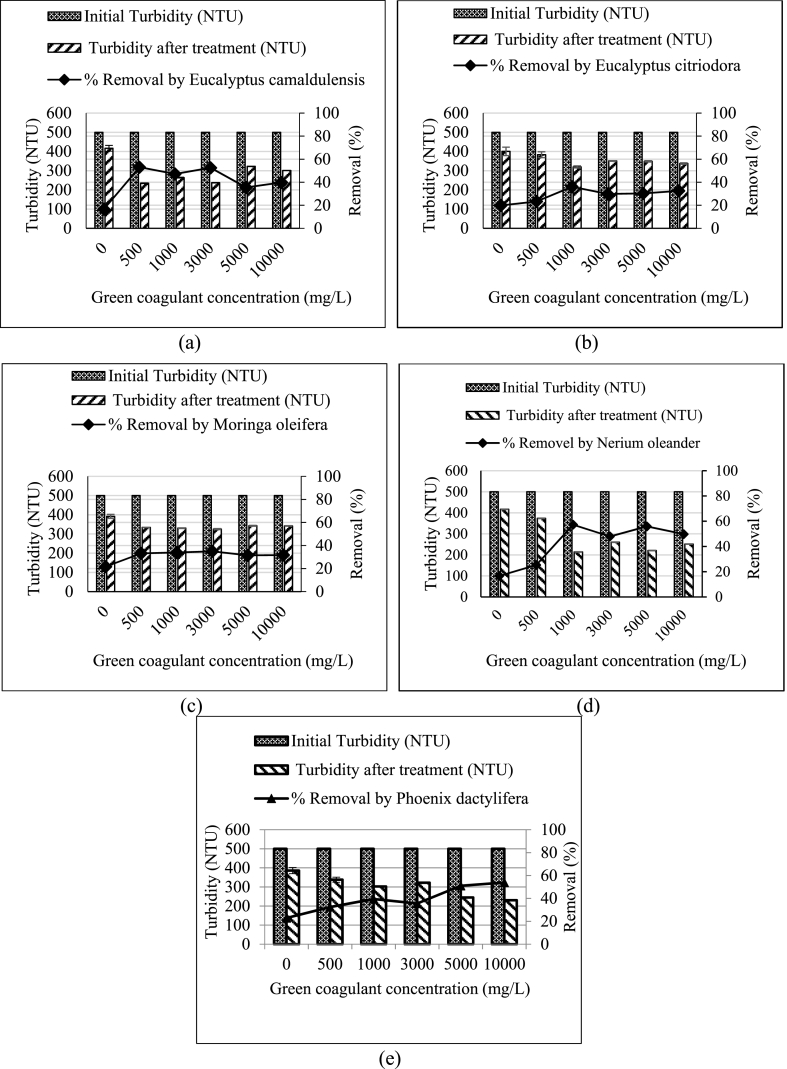
Role of the selected plants in turbidity removal with different coagulant dosages compared with the 500 NTU turbidity without the green coagulant as control (0 g/L): *Eucalyptus camaldulensis*, *Eucalyptus citriodora*, *Moringa oleifera*, *Nerium oleander* and *Phoenix dactylifera*.

The 13 local plant leaves were tested with the water extracted to remove turbidity. The turbidity removal of these leaves as coagulants are shown in Fig. 1, Fig. 2, Fig. 3. For *Acacia greggii* and *Moringa oleifera*, the maximum turbidity removal was reached to 45.5 and 33.2% respectively at a coagulant dosage of 500 mg/L (Fig. 1, Fig. 3). These results are the best dosages, because at 500 mg/L, a significant difference (p < 0.05) was observed compared with that at 1000–10,000 mg/L and compared with the control turbidity without added plant coagulants. For *Moringa oleifera*, another study tested the plant seed as water extraction coagulant with the best concentration of 70 mg/L that resulted in 63.7% turbidity removal (37 NTU in wastewater before treatment) from a wastewater from an oil refinery [30]. *Eucalyptus citriodora* and *Nerium oleander* had the best removal rates by using 1000 mg/L of green coagulant, reaching 36.1% and 57.2% removal efficiency (Fig. 3). Five green coagulants from *Aloe barbadensis*, *Bougainvillea glabra*, *Clerodendrum inerme*, *Dianthus caryophyllus* and *Eucalyptus camaldulensis* with 3000 mg/L concentration reached 67.9%, 43.3%, 47.6%, 52.6% and 52.5% removal efficiency (Fig. 1, Fig. 2). *Azadirachta indica, Conocarpus lancifolius* and *Phoenix dactylifera* at a concentration of 5000 mg/L reached 67.2%, 75.5% and 51.0% removal efficiency.

Green coagulant of *Albizia lebbeck (L.)* reached high removal efficiency (39.3%) at a concentration of 10,000 mg/L (Fig. 1) [19,25]. used *Azadirachta indica* to remove turbidity with removal efficiencies of 26.9% and 43.96% at concentrations of 10,000 and 1000 mg/L, respectively. By comparison, the concentration remarkably affected the removal efficiency, because 5000 mg/L *Azadirachta indica* green coagulant reached 67.2% removal efficiency. Table 2 summarises the best coagulant concentration and turbidity removal for the 13 tested plant-based coagulants.

Five plant leaves (*Aloe barbadensis, Bougainvillea glabra, Clerodendrum inerme, Dianthus caryophyllus and Eucalyptus camaldulensis*) with 3000 mg/L concentration reached the best turbidity removal rates of 67.9%, 43.3%, 47.6%, 52.6% and 52.5%, respectively (Table 3). At 5000 mg/L, *Azadirachta indica*, *Conocarpus lancifolius* and *Phoenix dactylifera* yielded good removal rates of 67.2%, 75.5% and 51.0% respectively. High (10,000 mg/L) and low concentrations (500 and 1000 mg/L) resulted in low turbidity removal of 33.2%–57.2%. The low efficiency removal for plant coagulant is caused by water extraction, which could not extract the proteins [27]. The turbidity removal rates of the seven plant leaves were higher than those of the six other plant leaves. This finding can be attributed to the role of the natural coagulants, which differ depending on the plant type and extraction methods [7]. [31] compared *Moringa* coagulant extracted using deionised water and 0.5 M NaCl solvent at dosages of 4 and 2 mg/mL dosage, and 37% and 91% turbidity removal efficiencies were obtained. The extraction of green coagulant by salt caused the cleavage of the protein–protein bond of the green coagulants [27,32].

**Table 3:** Turbidity removal of 13 locally selected plants.

Plant	Concentration (mg/L)	Removal %
*Acacia greggii*	500	45.5
*Albizia lebbeck (L.)*	10,000	39.3
*Aloe barbadensis*	3000	67.9
*Azadirachta indica*	5000	67.2
*Bougainvillea glabra*	3000	43.3
*Clerodendrum inerme*	3000	47.6
*Conocarpus lancifolius*	5000	75.5
*Dianthus caryophyllus*	3000	52.6
*Eucalyptus camaldulensis*	3000	52.5
*Eucalyptus citriodora*	1000	36.1
*Moringa oleifera*	500	33.2
*Nerium oleander*	1000	57.2
*Phoenix dactylifera*	5000	51

The overall result of turbidity removal from experiments was medium, because only extraction by water was carried out, and not all plant leaf compounds were extracted. Salt solution extraction has a much better efficiency than distilled water extraction.

### 3.2 Effect of green coagulant mass on removal efficiency

**Fig. 4:**
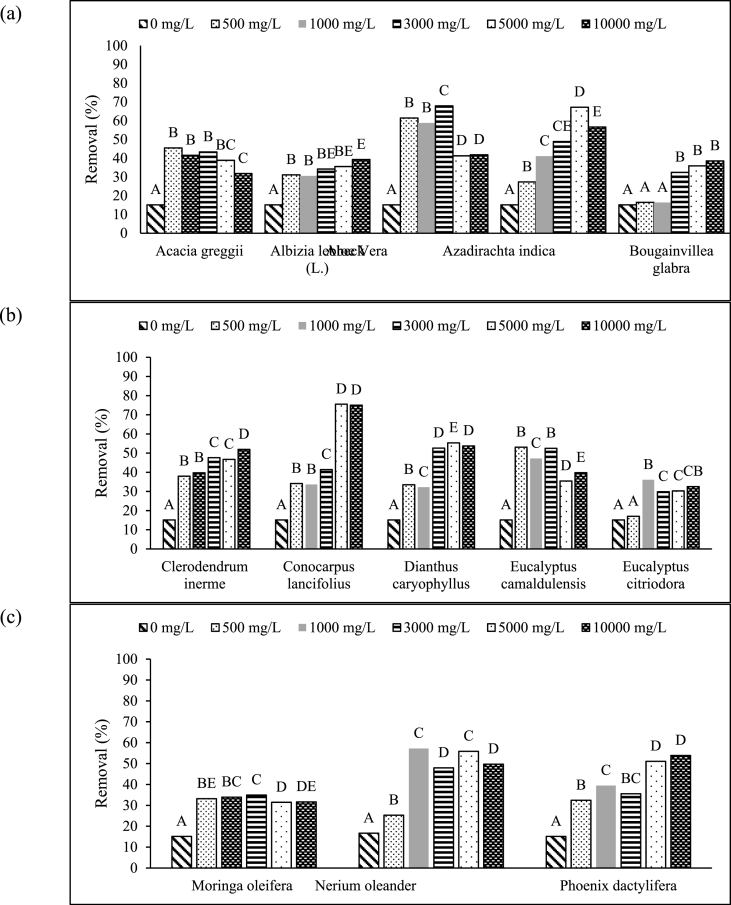
Statistical analysis for the effect of coagulant mass on turbidity removal for different plants. Different letters represent statistically significant differences in coagulant concentration for each plant to remove turbidity (*p* < 0.05).

The best concentration of green coagulant was determined via statistical analysis by using analysis of variance and post-hoc test by using Turkey HSD. All 13 plants can remove turbidity from water by using different coagulant concentrations. The best coagulant concentration for *Acacia g*r*eggii* and *Aloe barbadensis* was 500 mg/L with removal efficiencies of 45.47% and 61.4%, respectively, according to Turkey HSD analysis with *p* < 0.05 (Fig. 4(a)). *Bougainvillea glabra* was active in turbidity removal at a coagulant concentration of 3000 mg/L, and it reached a removal rate of 32.47%. Coagulant concentrations of 10,000 and 5000 mg/L are required for *Albizia lebbeck* (L.) and *Azadirachta indica* to remove 39.27% and 67.2% of water turbidity based on one-way ANOVA (*p* < 0.05, Fig. 4(a)) [19]. tested *Azadirachta indica* with 1000 mg/L green coagulant concentration and obtained 43.66% turbidity removal, which is consistent with the results obtained in our research for *Azadirachta indica* (Fig. 4(a)).

Statistical analysis for plants in Fig. 4(b) shown significant difference between different coagulant concentrations with *p* < 0.05. The required coagulant concentrations for *Clerodendrum inerme, Conocarpus lancifolius, Dianthus caryophyllus, Eucalyptus camaldulensis* and *Eucalyptus citriodora* are 10,000, 5,000, 3,000, 500 and 1000 with 51.9%, 75.5%, 52.6%, 53.1% and 36.1% removal efficiencies, respectively. Fig. 4(c) shows that at coagulant concentrations of 500, 1000 and 5000 mg/L for *Moringa oleifera*, *Nerium oleander* and *Phoenix dactylifera* resulted in 33.2%, 57.2% and 51.1% turbidity removal efficiencies based on Turkey HSD analysis at p < 0.05. After SPSS analysis, the best green coagulant concentrations were determined, as shown in Table 4. Seven plants with the best green coagulant masses were selected to be processed with future study application and more extraction methods. They can also be used in the recycling and recovery of waste from agriculture as green coagulants. The green coagulant from rice husk derived (75–250 mg/L) can be used for the treatment of both urban and agricultural runoffs with removal efficiencies for turbidity, TSS and COD of 85.85%–95.17%, 85.06%–89.82% and 68.54%–74.23%, respectively [33].

**Table 4:** Seven best local selected plants for turbidity removal.

Plant	Concentration (mg/L)	Removal (%)
*Albizia lebbeck (L.)*	10,000	39.3
*Azadirachta indica*	5000	67.2
*Clerodendron Inerme*	10,000	51.9
*Conocarpus lancifolius*	5000	75.5
*Dianthus caryophyllus*	3000	52.6
*Nerium oleander*	1000	57.2
*Phoenix dactylifera*	5000	51.1

According to many studies, plant-based coagulants perform well in lowering sludge residue after treatment by five times than using chemical coagulants as alum [34,35]. The chemicals such as alum will lead to increase in sludge residue which requires water molecules three times to obtain covalent bond [36]. The less sludge residue by plant-based coagulants becomes the evidence for the efficiency of sustainable green coagulant in wastewater the treatment.

## 4 Conclusions

The study involved the addition of the leaf powder water extracts as novel green coagulants for water decontamination applications. The suitable green coagulant concentrations showed differences between plants. Therefore, preliminary test is necessary to evaluate the optimum dosage. By screening 13 selected local Iraqi plants, *Conocarpus lancifolius*, *Azadirachta indica* and *Nerium oleander* had the highest turbidity removal efficiencies of 75.5% and 67.2% at a concentration of 5000 mg/L and 57.2% at a concentration of 1000 mg/L. The use of different extraction methods, such as salt (NaOH) and acid (HCl), is suggested in future studies to investigate the best performance extraction solution.

## Author contribution statement

Israa Al-Baldawi: Conceived and designed the experiments; Analyzed and interpreted the data; Wrote the paper. Ayat Khaled Salem: Performed the experiments; Contributed reagents, materials, analysis tools or data. Asia Fadhile Almansoory: Analyzed and interpreted the data; Contributed reagents, materials, analysis tools or data; Wrote the paper.

## Data availability statement

Data included in article/supp. material/referenced in article.

## Additional information

No additional information is available for this paper.

## Declaration of competing interest

The authors declare that they have no known competing financial inter- ests or personal relationships that could have appeared to influence the work reported in this paper.

Asia Fadhile Almansoory reports administrative support was provided by 10.13039/100012026University of Basrah. Israa Al-Baldawi reports a relationship with University of Baghdad that includes: employment.
